# Study for revision of Hwa-Byung Scale: The Delphi Method

**DOI:** 10.1192/j.eurpsy.2022.1405

**Published:** 2022-09-01

**Authors:** S. Lee, J. Kim, S.-A. Park, Y. Kwan, S.-W. Choi

**Affiliations:** 1 Duksung Women’s University, Clinical Psychology, Soul, Korea, Republic of; 2 Yonsei University Wonju College of Medicine, Psychiatry, Wonju, Korea, Republic of

**Keywords:** Oriental neuropsychiatry, Delphi method, Hwa-Byung, scale revision

## Abstract

**Introduction:**

Hwa-Byung is a mental syndrome classified as a “cultural-related syndrome” which reflects the cultural characteristics of Korea in DSM-IV. Hwa-Byung is caused by anger, which is characterized by feelings of anger or resent about unreasonable social violence and trauma. Kwon et al (2008) had developed self-report measure to assess severity of Hwa-Byung but it has several limitations to use in current clinical settings. Therefore, we investigated opinions of experts who have professionality in giving treatment of Hwa-Byung patients in the clinical settings.

**Objectives:**

The present study aimed to reach consent of oriental neuropsychiatrists’ opinions about the direction of revision of the Hwa-Byung scale.

**Methods:**

The Delphi method is a survey method that induces people to freely present their opinions without face-to-face processes and reaches consent through continuous feedback of survey results while ensuring anonymity. The Consensus Panel consists of 16 experts who are Oriental neuropsychiatrists and have experience in diagnosing patients with Hwa-Byung. A total of four surveys were conducted as consensus was reached on the fourth round. Each questionnaire was distributed by mail to a panel of experts and was asked to submit a response after receiving the questionnaire.

**Results:**

The results of the study are as follows. First, common factors for Hwa-Byung include anger, resent/blame, modify memory bias and attention bias for anger events. Second, characteristics of young Hwa-Byung patients include stress caused by social factors and excessive immersion in certain things such as drinking or smoking.

**Conclusions:**

Therefore, when revising the Hwa-Byung scale, it would be necessary to include these factors.

**Disclosure:**

No significant relationships.

